# Therapeutic monoclonal antibodies for diabetic kidney disease: a narrative review from basic mechanisms to clinical evidence

**DOI:** 10.3389/fendo.2026.1868198

**Published:** 2026-07-09

**Authors:** Xiu Hong Yang, Yao Liu, Chen Sheng Fu, Hui Min Jin, Zhi Bin Ye

**Affiliations:** 1 Department of Nephrology, Huadong Hospital, Fudan University, Shanghai, China; 2 Shanghai Key Laboratory of Clinical Geriatric Medicine, Huadong Hospital, Fudan University, Shanghai, China; 3 Shanghai Dong Ji Fresenius Hemodialysis Center, Shanghai, China; 4 Division of Nephrology, Shanghai Pudong Hospital, Fudan University, Shanghai, China

**Keywords:** clinical translation, diabetic kidney disease, monoclonal antibodies, PCSK9 inhibitors, renoprotection

## Abstract

**Introduction:**

Diabetic kidney disease (DKD) remains a leading cause of end-stage kidney disease despite advances with renin–angiotensin system blockade, sodium–glucose cotransporter 2 inhibitors, finerenone, and glucagon-like peptide-1 receptor agonists. Therapeutic monoclonal antibodies may offer a targeted strategy to modulate inflammatory, fibrotic, metabolic, and vascular pathways involved in DKD. This review summarizes the mechanistic rationale, clinical evidence, translational barriers, and future prospects of antibody-based therapies for DKD.

**Methods:**

We conducted a narrative literature review using PubMed, the Cochrane Library, and ClinicalTrials.gov from database inception to March 31, 2026. We prioritized peer-reviewed preclinical studies, clinical trials, and high-quality reviews addressing mAb-based strategies targeting DKD-related pathways, including TGF-β1, VEGF-B, CTGF, suPAR, integrin αvβ8, signal regulatory protein α, and PCSK9.

**Results:**

Phase II studies of anti-TGF-β1 and anti-VEGF-B antibodies failed to show meaningful renal benefit, highlighting challenges such as pathway redundancy, delayed intervention, insufficient intrarenal target engagement, and off-kidney toxicity. Anti-CTGF therapy showed an early signal of albuminuria, whereas anti-suPAR remains under clinical evaluation. Emerging preclinical targets, including integrin αvβ8 and signal regulatory protein α, may provide more kidney-focused modulation of fibrotic and inflammatory pathways. PCSK9 monoclonal antibodies, particularly evolocumab and alirocumab, appear promising because they may confer renal benefit through lipid lowering and kidney-intrinsic effects on lipotoxicity, oxidative stress, AMPK signaling, and profibrotic pathways.

**Conclusion:**

Monoclonal antibodies represent a biologically compelling but clinically underdeveloped strategy for DKD. Future progress will require earlier biomarker-informed patient selection, confirmation of intrarenal target engagement, appropriate renal endpoints, and rational combination with established kidney-protective therapies.

## Graphical Abstract

**Figure:**
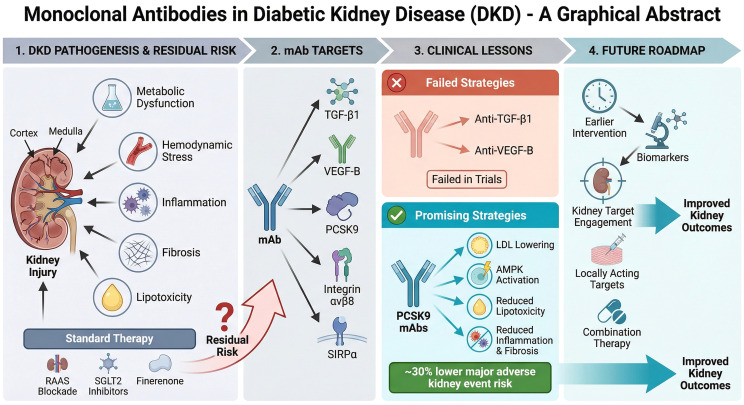

## 1 Introduction

Diabetic kidney disease (DKD) affects 20–40% of patients with diabetes and remains a leading cause of end-stage renal disease (1). Despite guideline-directed use of renin-angiotensin-aldosterone system (RAAS) blockers, sodium-glucose cotransporter-2 (SGLT2) inhibitors, and mineralocorticoid receptor antagonists (MRAs), a substantial residual risk of progressive kidney function decline persists (2). This unmet need has driven the search for therapies that intervene more precisely in the underlying molecular pathways—an opportunity that therapeutic mAbs are uniquely positioned to fulfill.

Monoclonal antibodies (mAbs) have revolutionized the treatment of cancer, autoimmune diseases, and hyperlipidemia (3–5). Beyond these established indications, mAbs have evolved from experimental reagents into a major class of precision biologic therapeutics (6, 7). Their clinical value derives from several pharmacological and biological advantages, including high target specificity, predictable target engagement, prolonged systemic half-life, and the capacity to neutralize soluble mediators, block receptor–ligand interactions, deplete pathogenic cell populations, or deliver therapeutic payloads to selected tissues. Over the past decade, advances in antibody engineering have further broadened this field from conventional IgG antibodies to humanized and fully human antibodies, Fc-engineered antibodies, bispecific antibodies, antibody–drug conjugates, and antibody fragments. These innovations have established mAbs as one of the most rapidly expanding categories of biologic drugs.

In nephrology, their application has been largely confined to primary glomerular diseases (8). More broadly, antibody-based therapies have also been used or explored in kidney transplantation, complement-mediated kidney disorders, and other immune-mediated renal conditions. However, a growing understanding of DKD as an inflammatory, fibrotic, and metabolically driven condition has opened new opportunities for biologic intervention (9). DKD is not a classical antibody-mediated disease; rather, its progression is driven by intersecting metabolic, hemodynamic, inflammatory, fibrotic, and vascular pathways. This biological complexity makes DKD a challenging but potentially attractive setting for antibody-based intervention. In particular, mAbs may be suitable when pathogenic mediators are extracellular, ligand-driven, or cell-surface accessible, such as TGF-β-related fibrosis, VEGF-B-mediated lipid handling, suPAR signaling, integrin-dependent TGF-β activation, SIRPα-mediated inflammatory crosstalk, and PCSK9-driven dyslipidemia and lipotoxicity.

**Figure 1:**
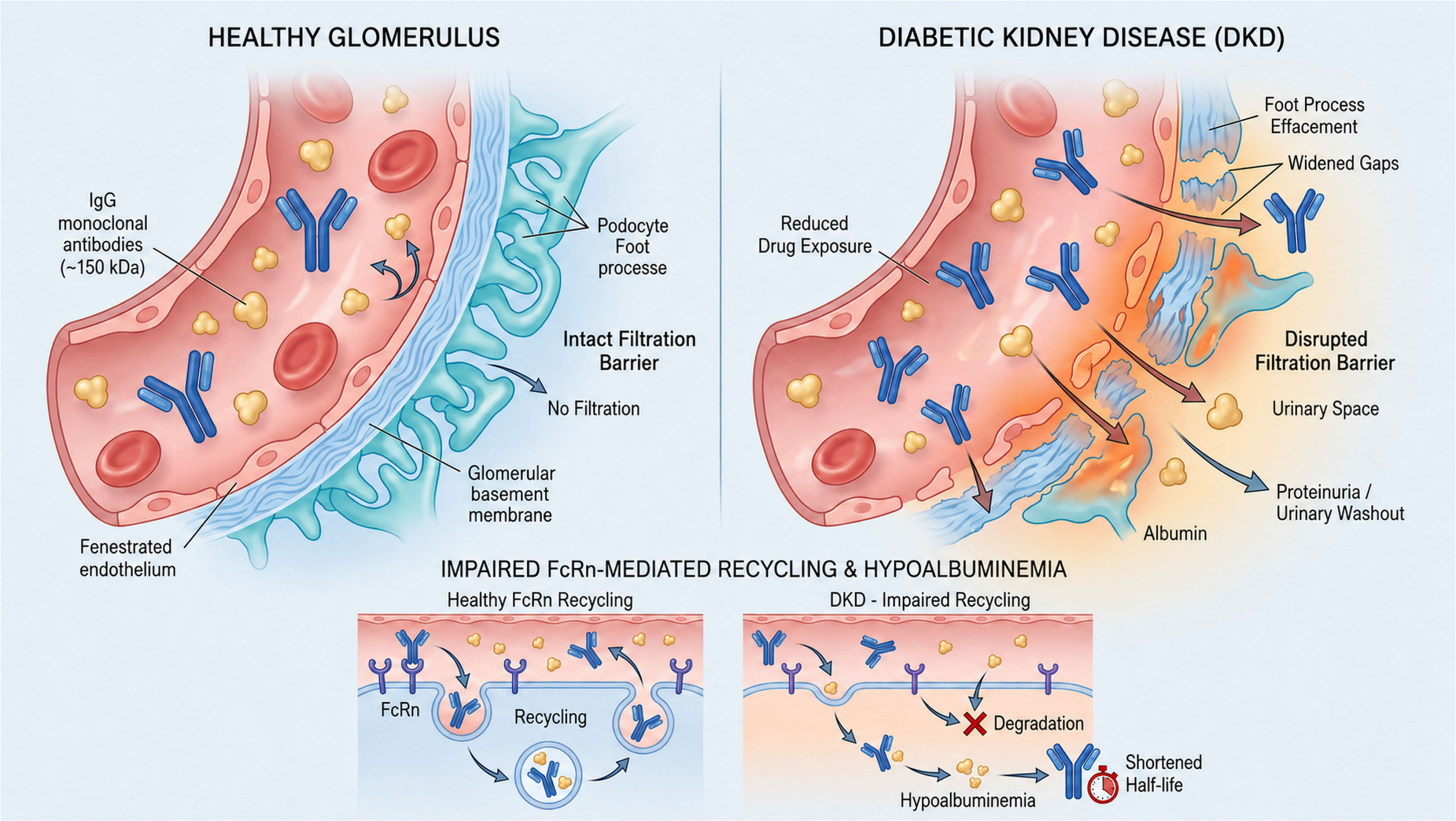
Altered pharmacokinetic and pharmacodynamic behavior of therapeutic mAbs in DKD. In the healthy kidney, the intact glomerular filtration barrier prevents the filtration of large IgG-based monoclonal antibodies. In DKD, podocyte injury, foot process effacement, and barrier disruption permit urinary leakage of these molecules, potentially reducing systemic exposure and renal target engagement. Hypoalbuminemia and impaired FcRn-mediated recycling may further shorten antibody half-life. These disease-related changes help explain why mAb disposition and efficacy in DKD may differ substantially from those in individuals with preserved glomerular barrier integrity.

This review traces the journey of mAbs for DKD from foundational discovery to clinical testing, highlighting early translational failures, emerging preclinical targets (αvβ8, SIRPα), and the dual-benefit paradigm of PCSK9 inhibition. We conclude by proposing concrete strategies for future trial design and biomarker-guided patient selection to accelerate the arrival of effective biologic therapies for DKD. **Figure 1** provides an overview of the scope and conceptual framework of this review.

## 2 Search strategy

This narrative review was based on a literature search of PubMed, the Cochrane Library, and ClinicalTrials.gov from database inception to March 31, 2026. Search terms included “diabetic kidney disease,” “diabetic nephropathy,” “monoclonal antibody,” “biologic therapy,” “TGF-β,” “VEGF-B,” “CTGF,” “suPAR,” “integrin αvβ8,” “SIRPα,” “PCSK9,” “evolocumab,” and “alirocumab.” We prioritized peer-reviewed original studies, clinical trials, systematic or high-quality narrative reviews, and key preclinical studies directly relevant to antibody-based interventions in DKD. Studies were included if they addressed the mechanistic rationale, pharmacology, preclinical efficacy, clinical outcomes, safety, or translational barriers of mAb-based strategies in DKD or closely related kidney disease contexts. Non-English articles without accessible English abstracts, studies not relevant to DKD or antibody-based therapy, and publications lacking sufficient methodological or mechanistic detail were excluded.

## 3 Pathophysiological basis for mAbs therapy in DKD

**Figure 2:**
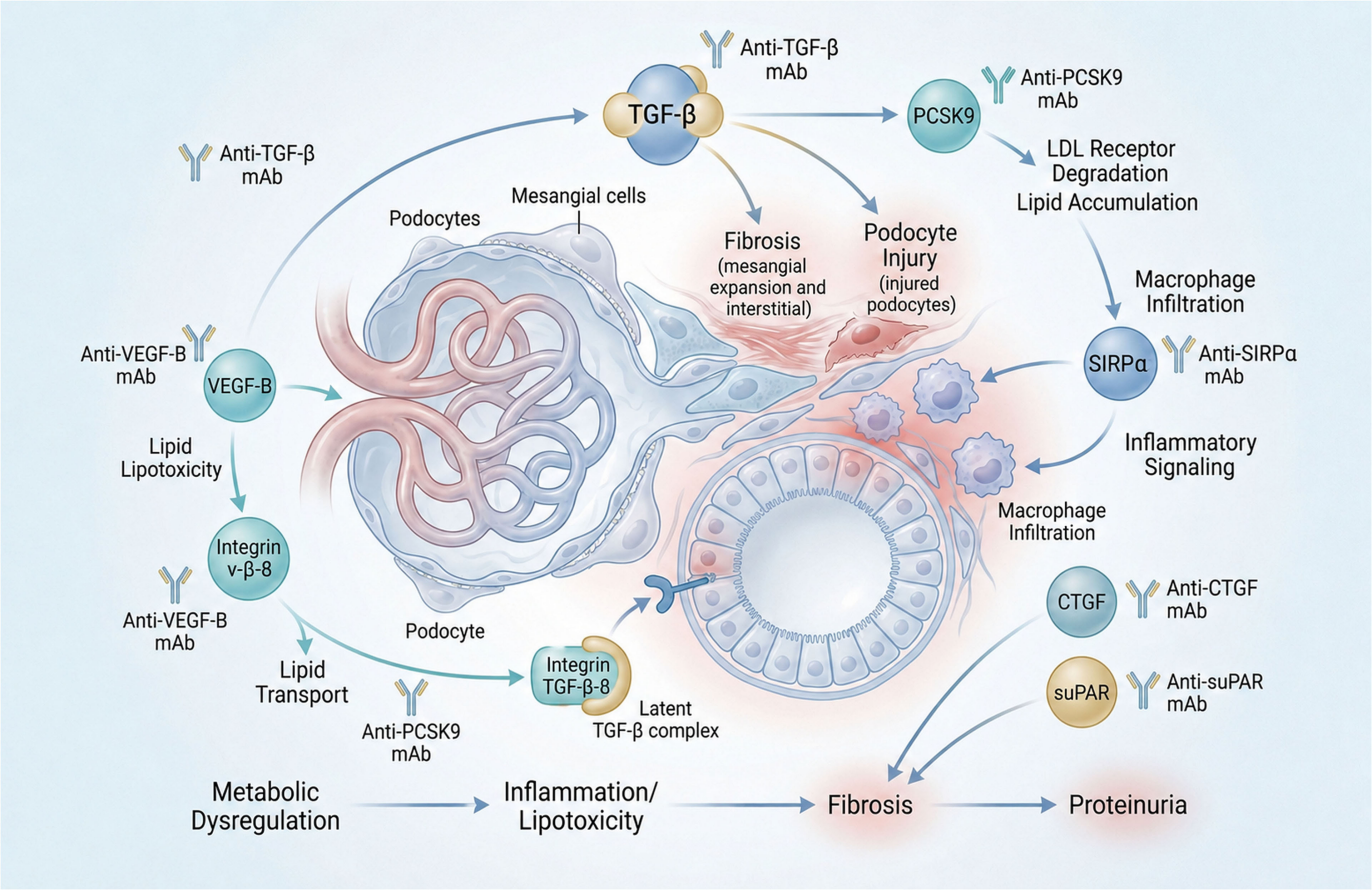
Therapeutic target landscape of mAbs in DKD. This schematic summarizes the major molecular pathways that support mAb development in DKD. DKD is driven by intersecting fibrotic, inflammatory, and metabolic mechanisms involving podocytes, mesangial cells, tubular epithelial cells, and infiltrating immune cells. Key therapeutic targets include TGF-β and integrin αvβ8 in profibrotic signaling, VEGF-B and PCSK9 in renal lipid dysregulation, and SIRPα in immune-cell recruitment and inflammatory crosstalk. CTGF and suPAR are shown as additional candidate targets. Together, these pathways illustrate the rationale for precision biologic intervention in DKD.

The rationale for mAb therapy in DKD depends not only on whether a pathway contributes to disease progression, but also on whether the pathogenic mediator is accessible to antibody-based intervention. Recent kidney-disease reviews have emphasized extracellular and compartment-specific mechanisms, including ligand–receptor interactions, extracellular matrix remodeling, and inflammatory crosstalk, as useful frameworks for identifying druggable targets (10). The pathogenesis of DKD is multifactorial and involves hemodynamic alterations, metabolic dysregulation, inflammation, and fibrosis. These processes interact as an integrated molecular network rather than isolated pathways, providing a rationale for targeted interventions against key extracellular or cell-surface mediators (11). The major therapeutic targets currently under investigation in DKD are summarized in **Figure 2**. Key pathways targeted by mAbs include the following:

### 3.1 The Transforming growth factor-beta pathway

TGF-β is a master regulator of fibrosis and has long been regarded as a central driver of DKD (12). However, systemic inhibition has proven difficult because of its pleiotropic roles in immune regulation and tissue homeostasis. Neutralizing mAbs against TGF-β1 were designed to suppress this profibrotic axis, but clinical translation has been limited by pathway redundancy, late intervention in patients with established fibrosis, and the potential risk of disrupting physiological TGF-β functions. A key unresolved question is whether more localized modulation of TGF-β activation, such as targeting integrin-mediated activation within the renal microenvironment, can preserve antifibrotic benefit while avoiding systemic toxicity.

### 3.2 The vascular endothelial growth factor family

VEGF-A is essential for podocyte function, but its inhibition may paradoxically aggravate thrombotic microangiopathy (13). This illustrates an important principle for antibody therapy in DKD: not all disease-associated pathways are suitable for systemic blockade, because some also maintain glomerular microvascular integrity (13). Moreover, the frequent coexistence of diabetes and hypertension further complicates renal vascular biology. Diabetic and hypertensive kidney injury share vascular mechanisms, including endothelial dysfunction, altered capillary density, impaired angiogenic signaling, and changes in VEGF-related regulatory pathways (14). By contrast, VEGF-B has emerged as an alternative target because of its role in renal lipid transport.

### 3.3 Lipid metabolism and PCSK9

Proprotein convertase subtilisin/kexin type 9 (PCSK9) promotes degradation of low-density lipoprotein receptors (LDLR), thereby contributing to dyslipidemia and renal lipid accumulation. Beyond lipid regulation, PCSK9 has also been implicated in direct renal injury through inflammatory and profibrotic signaling pathways (15). In DKD, lipid deposition in podocytes, mesangial cells, and tubular epithelial cells may promote oxidative stress, mitochondrial dysfunction, inflammation, and fibrosis. PCSK9 mAbs such as evolocumab and alirocumab primarily reduce LDL-C, but experimental and clinical observations suggest potential renal benefits through attenuation of lipotoxic and inflammatory–fibrotic signaling. The main unresolved issue is whether these renal effects are independent of lipid lowering. Dedicated DKD trials are needed to determine whether PCSK9 mAbs can slow kidney disease progression beyond cardiovascular risk reduction.

### 3.4 Immune and inflammatory targets

In DKD, increasing evidence has identified specific immune and inflammatory mediators as important contributors to disease progression. Among these, signal regulatory protein α (SIRPα) and integrins such as αvβ8 have emerged as novel targets. SIRPα, which is classically involved in the regulation of macrophage phagocytosis, appears to modulate immune-cell infiltration into the renal interstitium. Meanwhile, integrin αvβ8 has been shown to locally activate TGF-β, a key profibrotic cytokine, within the diabetic renal microenvironment. Together, these molecules contribute to the interplay between immune-cell recruitment and fibrotic signaling and therefore represent promising therapeutic targets in DKD (16, 17). SIRPα may regulate immune-cell infiltration and immune–metabolic crosstalk, whereas αvβ8 locally activates latent TGF-β within the renal microenvironment. mAbs targeting these pathways may therefore provide more spatially restricted modulation of inflammation and fibrosis than systemic cytokine blockade. However, these approaches remain largely preclinical, and their long-term effects on host defense, tissue repair, and immune tolerance require further evaluation before clinical translation.

## 4 From preclinical promise to clinical reality

### 4.1 Preclinical evidence of emerging targets

Preclinical studies have provided the mechanistic basis for several emerging mAb-based strategies in DKD, mainly involving antifibrotic signaling, immune-inflammatory crosstalk, and kidney-directed molecular delivery. Among these targets, integrin αvβ8 is of particular interest because it locally activates latent TGF-β within the renal microenvironment; therefore, αvβ8 inhibition may suppress profibrotic signaling in a more spatially restricted manner than systemic TGF-β blockade. SIRPα neutralization has also shown potential by improving renal fatty acid metabolism and reducing proteinuria, polyuria, and kidney dysfunction despite persistent hyperglycemia. In addition, exploratory approaches such as p75NTR targeting and anti-VEGFR2 F(ab’)2–SS31 conjugates suggest that antibody-based strategies may be used not only for target neutralization but also for kidney-directed delivery of protective payloads. However, most evidence remains limited to animal models with early intervention and short follow-up, and translation to patients with established DKD remains uncertain. Key preclinical studies are summarized in **Table 1** (18–21).

**Table 1:** Preclinical studies of mAbs in DKD.

Target/Drug name	Research stage	Key results	Source
αvβ8 integrin/MEDI8367	Preclinical	In CKD models (including DKD), this antibody alleviated renal fibrosis, reduced renal damage, and improved renal function by inhibiting TGF-β activation.	Kidney360–2025 (18)
SIRPα/Neutralizing antibody	Preclinical	In a type 1 diabetes mouse model, SIRPα neutralization improved fatty acid metabolism in the kidneys, alleviated proteinuria and polyuria, and reduced serum creatinine.	JASN 2025 (19)
p75NTR/anti-proNGF mAb	Preclinical	In STZ-induced diabetic mice, treatment partially reversed microRNA and mRNA expression changes associated with diabetic nephropathy and improved plasma urea and creatinine.	Gene 2022 (20)
VEGFR2/Antibody-drug conjugate	Preclinical	Anti-VEGFR2 F(ab’)2 fragment conjugated to SS31 peptide showed enhanced renal accumulation and alleviated glomerular structural damage, inflammation, and fibrosis in DKD mouse models.	Nat Commun 2023 (21)

### 4.2 Clinical trials: lessons from failure and partial success

Early clinical trials of mAbs in DKD have yielded mixed results. The anti-TGF-β1 antibody LY2382770 was among the first mAbs to enter phase II testing. Although preclinical studies showed antifibrotic activity, the trial was terminated early for futility; in 416 patients with DKD, LY2382770 failed to slow eGFR decline compared with placebo despite an acceptable safety profile (22). Similarly, the anti-VEGF-B antibody CSL346 was evaluated in a phase II trial involving 114 patients with DKD but did not reduce UACR, the primary endpoint. Higher doses were also associated with increased diastolic blood pressure, raising concern about on-target but off-kidney vascular effects (23).

Other mAb strategies have shown partial or still-emerging signals. The anti-CTGF antibody FG-3019 was evaluated in a phase I study of 24 patients with diabetes and microalbuminuria and was well tolerated, with mean UACR decreasing from 48 mg/g before treatment to 20 mg/g on day 56 (24). Anti-suPAR therapy remains under clinical evaluation; a phase II basket trial of WAL0921, including patients with DKD, completed enrollment of the first DKD dose cohort in September 2024, but results have not yet been reported (25).

Several lessons can be drawn from these trials. For antifibrotic strategies such as TGF-β1 blockade, intervention may have occurred too late, when glomerulosclerosis and tubulointerstitial fibrosis were already established. In addition, pathway redundancy may have limited efficacy because multiple profibrotic mediators, including CTGF, PDGF, and IL-11, can remain active despite blockade of a single cytokine. For VEGF-B blockade, systemic exposure did not necessarily ensure intrarenal target engagement, and dose escalation was limited by vascular safety signals. These findings suggest that future trials should enroll biomarker-enriched patients earlier in the disease course, confirm renal target engagement, use endpoints appropriate to the expected biological effect, and carefully monitor off-kidney toxicity.

## 5 PCSK9 monoclonal antibodies: a paradigm shift

Among recent advances, PCSK9-targeting mAbs appear particularly promising because they may confer both cardiovascular and renal benefits.

### 5.1 Mechanisms beyond lipid lowering

PCSK9 inhibitors such as evolocumab and alirocumab are well established for their potent low-density lipoprotein cholesterol (LDL-C)-lowering effects. More recent basic research has suggested additional direct renoprotective mechanisms. In db/db mouse models of DKD, PCSK9 inhibition with the small-molecule inhibitor SBC-115076 activated the AMPK-PGC-1α-FoxO3a signaling pathway, a key regulator of cellular energy homeostasis (26). This activation reduced renal lipotoxicity, oxidative stress, and apoptosis, and was accompanied by a significant decrease in UACR and improvement in renal histology.

In addition, PCSK9 may contribute to renal fibrosis indirectly. By downregulating LDL receptors, it promotes lipid accumulation in mesangial and tubular cells, thereby triggering inflammatory and profibrotic responses. PCSK9 blockade has also been reported to reduce angiopoietin-like 3 (Angptl3) expression and inhibit TGF-β signaling, suggesting multiple parallel pathways through which renal protection may be achieved (27).

### 5.2 Clinical evidence: real-world and *post-hoc* outcomes

Although no dedicated large-scale randomized controlled trial (RCT) of PCSK9 inhibitors in DKD has yet been completed, accumulating real-world and *post hoc* evidence is noteworthy. The main clinical evidence for PCSK9 monoclonal antibodies in DKD is summarized in **Table 2**.

- TriNetX real-world analysis: In 40,978 patients with type 2 diabetes and dyslipidemia, use of PCSK9 inhibitors (alirocumab or evolocumab) was associated with a 30% relative risk reduction in major adverse kidney events (MAKE; composite of ≥50% eGFR decline, end-stage kidney disease, or all-cause mortality; HR 0.70, 95% CI 0.61–0.81) (28).
- FOURIER trial (evolocumab): In 27,564 patients with atherosclerotic cardiovascular disease, approximately 37% of whom had diabetes, evolocumab reduced LDL-C by 59%. Exploratory analyses showed a nonsignificant trend toward reduced kidney failure (HR 0.80, 95% CI 0.61–1.05), but a significant reduction in the composite of death or kidney failure (HR 0.81, 95% CI 0.68–0.96) (29).
- ODYSSEY OUTCOMES trial (alirocumab): In 18,924 patients following acute coronary syndrome, alirocumab reduced major adverse cardiovascular events. Among those with chronic kidney disease at baseline (eGFR 30–60 mL/min/1.73 m²), alirocumab was associated with a slower decline in eGFR than placebo (difference +1.1 mL/min/1.73 m² per year; p=0.02) (30).
- Mendelian randomization studies: Genetic variants that mimic PCSK9 inhibition have been associated with slower eGFR decline and lower risk of DKD, although whether these renal effects are independent of LDL-C lowering remains uncertain (31).

Despite these encouraging observations, the available evidence is derived largely from *post hoc* analyses of cardiovascular trials or retrospective real-world studies. Dedicated prospective RCTs using renal composite outcomes as primary endpoints in a DKD-specific population are urgently needed to define the true magnitude of benefit.

### 5.3 Safety and positioning relative to current standard of care

According to clinical trial data, the most common adverse events associated with PCSK9 inhibitors are injection-site reactions (approximately 5%–7%) and nasopharyngitis (approximately 10%–11%). Unlike statins, PCSK9 inhibitors have not been associated with an increased risk of new-onset diabetes or deterioration in glycemic control (32).

**Figure 3:**
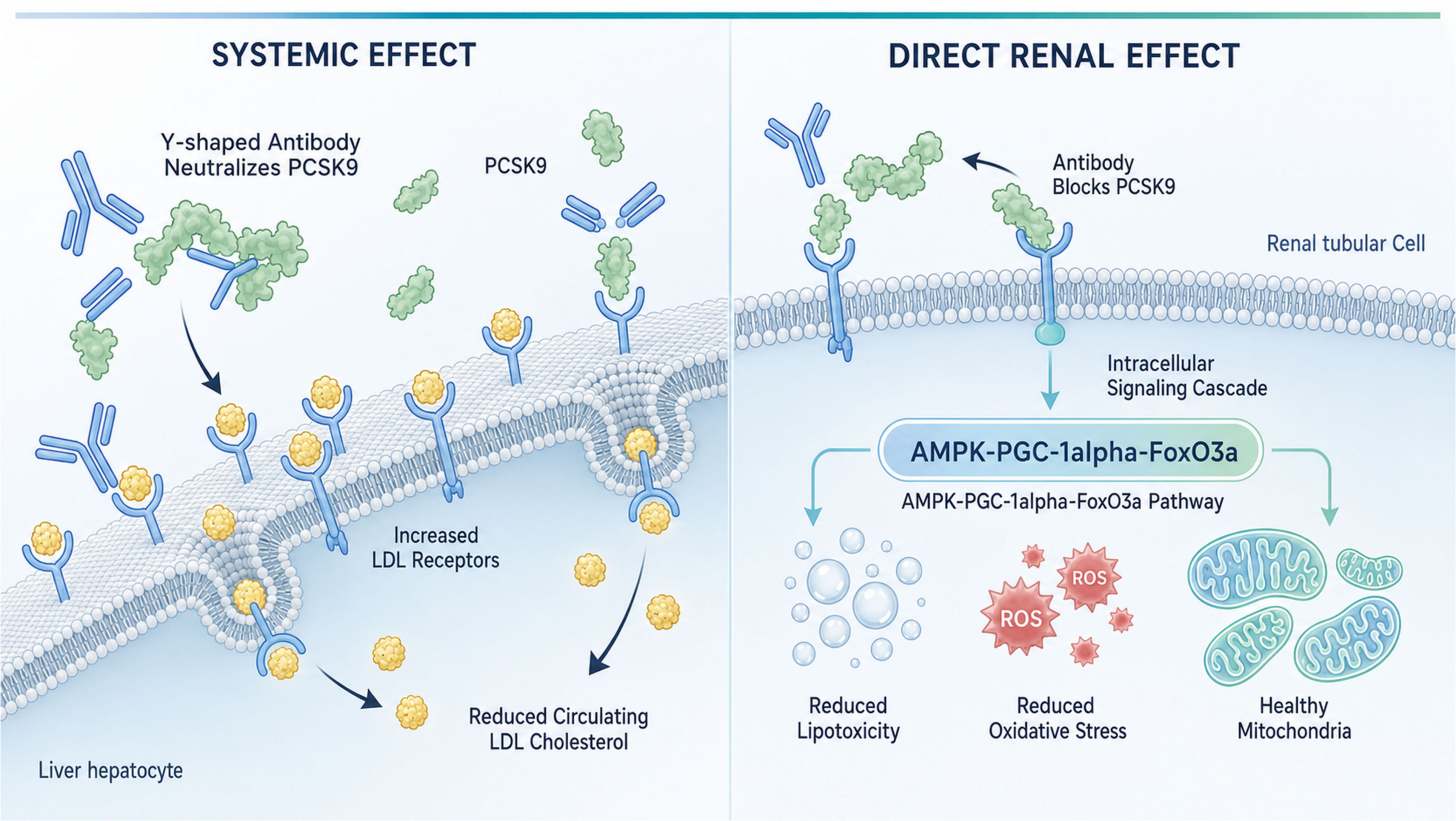
PCSK9 mAbs as a paradigm shift in DKD. PCSK9 inhibitors may benefit DKD through both systemic and kidney-intrinsic mechanisms. In the liver, mAb blockade of PCSK9 increases LDL receptor abundance and lowers circulating LDL cholesterol. In the kidney, experimental data suggest that PCSK9 inhibition may activate the AMPK–PGC-1α–FoxO3a axis, thereby reducing lipotoxicity, oxidative stress, apoptosis, and fibrotic signaling while preserving mitochondrial health. This dual mechanism distinguishes PCSK9 mAbs from earlier DKD biologic strategies and supports their emerging role as add-on therapy in high-risk patients.

Current first-line therapies for DKD include renin–angiotensin–aldosterone system (RAAS) blockers, sodium-glucose cotransporter 2 (SGLT2) inhibitors, finerenone, and glucagon-like peptide-1 receptor agonists (GLP-1 RAs). These agents reduce albuminuria and slow eGFR decline but do not abolish residual risk. mAbs are therefore not intended to replace standard therapy, but rather to be added in selected patients who remain at high risk, such as those with persistent UACR >300 mg/g despite maximally tolerated SGLT2 inhibitor and finerenone therapy (32). The potential synergy between PCSK9 mAbs and SGLT2 inhibitors is especially attractive: SGLT2 inhibitors improve glomerular hemodynamics and reduce inflammation, whereas PCSK9 mAbs may target lipotoxicity and fibrosis. At present, the dual systemic and intrarenal mechanisms supporting PCSK9 inhibition in DKD are summarized in **Figure 3**.

## 6 Pharmacokinetic and pharmacodynamic considerations of mAbs in DKD

The pharmacokinetic (PK) and pharmacodynamic (PD) behavior of therapeutic mAbs in patients with DKD deserves specific consideration because renal structural damage may alter both systemic exposure and intrarenal target engagement. Most IgG-based mAbs are large molecules of approximately 150 kDa and are therefore not freely filtered through an intact glomerular filtration barrier. In DKD, however, podocyte injury, glomerular basement membrane remodeling, and disruption of size- and charge-selective filtration may permit urinary leakage of IgG or IgG-like molecules. This loss may reduce circulating exposure, shorten effective half-life, and compromise drug availability at renal targets, particularly in patients with heavy proteinuria or nephrotic-range albuminuria.

Several disease-related factors may further modify mAb disposition in DKD. Hypoalbuminemia and impaired neonatal Fc receptor (FcRn)-mediated recycling may reduce IgG salvage and accelerate antibody clearance. Expanded extracellular volume, edema, and systemic inflammation may also influence the apparent volume of distribution and target-mediated drug disposition. These changes are particularly relevant for antibodies directed against soluble ligands, such as TGF-β1 or VEGF-B, because adequate systemic exposure does not necessarily guarantee sufficient intrarenal target engagement. Conversely, antibodies targeting circulating proteins, such as PCSK9, may be less dependent on renal tissue penetration, which may partly explain their more favorable translational profile.

From a PD perspective, DKD presents several additional challenges. First, pathway redundancy may blunt the biological effect of blocking a single ligand or receptor, especially in advanced fibrotic disease. Second, renal target engagement is rarely confirmed directly in clinical trials, and surrogate markers such as serum drug concentration or short-term UACR change may not fully reflect intrarenal pharmacological activity. Third, the optimal dose and dosing interval of mAbs in patients with severe proteinuria remain uncertain, because most approved antibodies are administered at fixed doses without adjustment for estimated glomerular filtration rate. Therefore, future DKD trials should incorporate PK sampling, anti-drug antibody monitoring, urinary or plasma biomarkers of target engagement, and prespecified analyses according to baseline proteinuria and kidney function. The key PK features of representative mAbs are summarized in **Table 3**.

**Table 2:** Summary of clinical evidence for PCSK9 mAbs in DKD.

Source	Drug	Population	Renal endpoint	Effect size (vs control)
FOURIER renal substudy	Evolocumab	ASCVD + diabetes	Composite of death/ESKD	HR 0.81 [0.68–0.96]
ODYSSEY renal substudy	Alirocumab	ACS + CKD (eGFR 30–60)	eGFR slope	+1.1 mL/min/1.73 m²/year
TriNetX real-world	Evolocumab/alirocumab	T2D + dyslipidemia	MAKE (≥50% eGFR drop, ESKD, death)	HR 0.70 [0.61–0.81]

**Table 3:** Pharmacokinetic features of selected mAbs in chronic kidney disease.

mAb (target)	Molecular format	Half-life (normal)	PK alterations in DKD	Proposed dose adjustment
Anti-TGF-β1	IgG4	12–15 days	May increase clearance in proteinuric DKD	None established
CSL346 (VEGF-B)	IgG1	18–21 days	Unknown	None
MEDI8367 (αvβ8)	IgG4	14–18 days (estimated)	Not studied	–
Evolocumab (PCSK9)	IgG2	11–17 days	No significant change reported in real-world studies	No adjustment
Alirocumab (PCSK9)	IgG1	17–20 days	Similar to general population	No adjustment

## 7 Safety and tolerability of mAbs in DKD

Although mAbs are generally well tolerated, several safety signals have emerged from DKD trials. Immunogenicity, particularly the development of anti-drug antibodies, may lead to infusion reactions and reduced efficacy. In the anti-TGF-β1 trial, no significant immunogenicity was reported, although long-term data remain limited. On-target but off-kidney effects continue to be a major concern (30). For example, VEGF-B blockade was associated with dose-dependent increases in diastolic blood pressure, possibly reflecting effects on vascular tone (33).

Anti-αvβ8 integrin antibodies may carry a theoretical risk of impaired mucosal immunity because of systemic modulation of TGF-β; however, preclinical studies suggest a favorable safety profile with predominantly kidney-restricted activity (34). In clinical practice, monitoring should include surveillance for allergic reactions, infection, and, in the case of anti-VEGF agents, hypertension and worsening proteinuria.

## 8 Economic and access considerations

The high cost of mAbs, for example approximately US$5,000–7,000 per year for evolocumab in the United States, remains a major barrier to widespread use in DKD (35), a highly prevalent condition that often requires long-term treatment. To date, no formal cost-effectiveness analysis has been performed specifically for DKD. Based on available real-world data, the number needed to treat to prevent one major adverse kidney event is estimated to be approximately 30–40, suggesting that the incremental cost-effectiveness ratio (ICER) may exceed commonly accepted thresholds unless drug prices decline. Biosimilars, as seen with agents such as adalimumab and rituximab, could reduce costs, but no biosimilar PCSK9 inhibitor is currently available for DKD indications. Potential prioritization strategies may include restricting mAb therapy to patients with rapid eGFR decline (>5 mL/min/1.73 m² per year) or to those carrying high-risk genetic variants, such as APOL1 risk genotypes in African American populations.

## 9 Barriers to translational success: Why have mAbs failed in DKD so far?

The failures of anti-TGF-β1 and anti-VEGF-B antibodies offer several important lessons.

- Limitations of animal models: Streptozotocin (STZ)-induced diabetes in rodents does not fully recapitulate human DKD, particularly the slow progression of fibrosis. Early intervention in mice may therefore poorly reflect treatment in patients with established disease.
- Target redundancy: Inhibition of TGF-β1 alone is unlikely to be sufficient because multiple profibrotic pathways, including CTGF, PDGF, and IL-11, remain active (36–38). Combination approaches may therefore be necessary.
- Timing of intervention: Once glomerulosclerosis and tubulointerstitial fibrosis are established, reversal with a single mAb is improbable. Future trials should prioritize patients with preserved renal function and substantial albuminuria.
- Surrogate endpoints: Early-phase studies frequently use change in UACR as a primary endpoint, but UACR may not reliably predict long-term eGFR slope. Regulatory agencies now recognize eGFR slope as a valid endpoint in DKD trials (39).
- Insufficient target engagement: For mAbs directed against soluble ligands such as VEGF-B, dose selection based solely on serum levels may not accurately reflect intrarenal tissue exposure (23).

**Figure 4:**
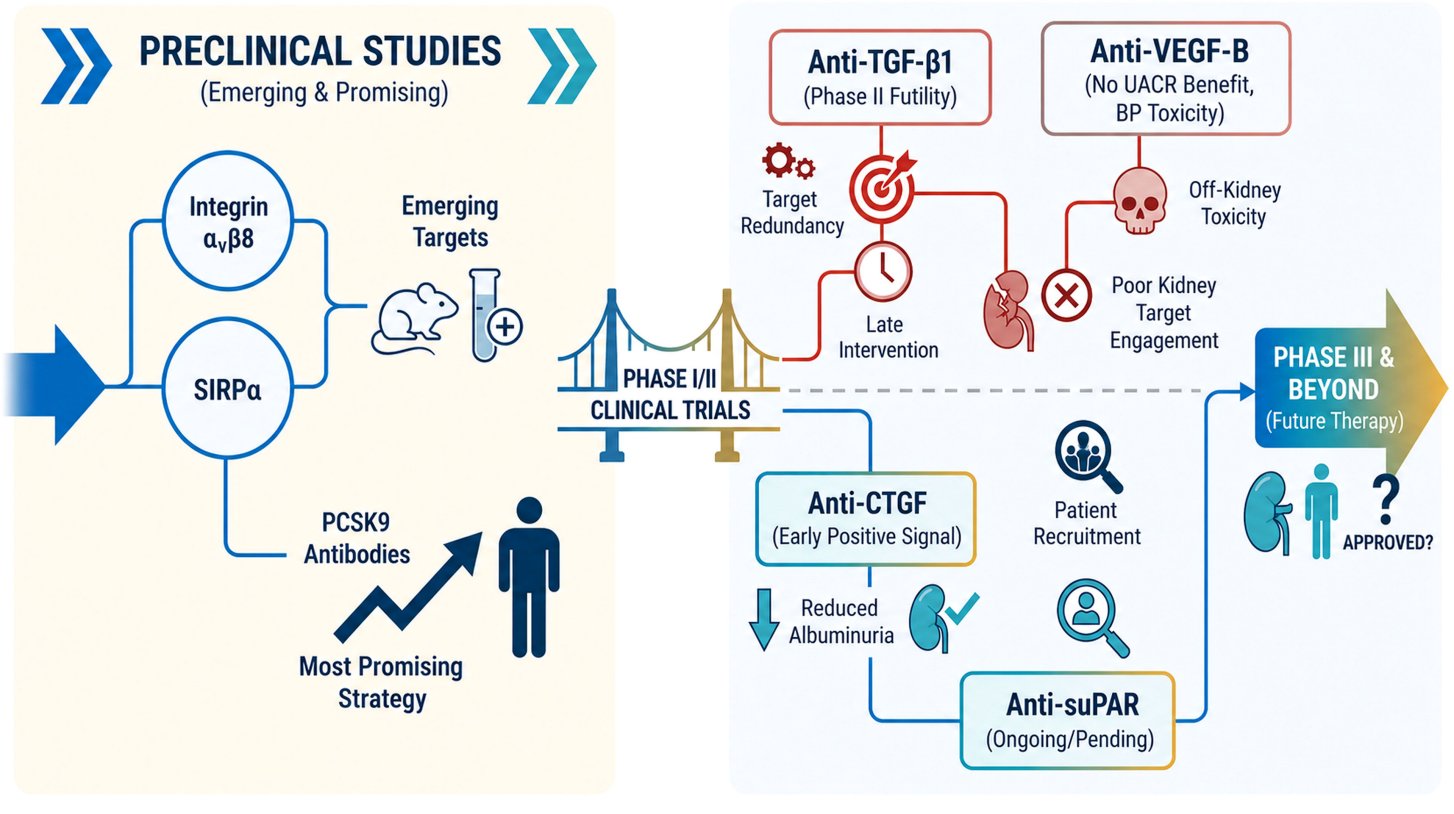
From preclinical promise to clinical reality in mAb therapy for DKD. This figure outlines the translational trajectory of mAb programs in DKD. Anti-TGF-β1 and anti-VEGF-B antibodies entered clinical testing on the basis of strong preclinical rationale but failed to deliver meaningful renal benefit in phase II studies. In contrast, anti-CTGF showed an early signal for albuminuria reduction, while anti-suPAR remains under clinical evaluation. Integrin αvβ8 and SIRPα represent emerging preclinical targets with kidney-focused mechanistic appeal. PCSK9 monoclonal antibodies currently represent the most promising translational direction, supported by both mechanistic and clinical outcome signals. Major barriers highlighted include target redundancy, intervention too late in disease course, insufficient kidney target engagement, and off-kidney toxicity.

These barriers have shifted attention toward either locally acting targets, such as αvβ8 integrin, or multifunctional targets, such as PCSK9, as summarized in **Figure 4**.

## 10 Future perspectives

The future of mAb therapy in DKD will likely depend on progress in several key areas.

### 10.1 Combination strategies

Simultaneous targeting of metabolic, inflammatory, and fibrotic pathways may produce synergistic therapeutic effects. For example, combining a PCSK9 mAb with an SGLT2 inhibitor could provide complementary metabolic and hemodynamic protection (40).

### 10.2 Novel antibody formats

Antibody-drug conjugates (ADCs) capable of delivering antifibrotic or antioxidant payloads specifically to the kidney, such as anti-VEGFR2 F(ab’)2 fragments conjugated to SS31 peptides, may improve efficacy while reducing off-target toxicity (21). In addition, bispecific antibodies targeting pathways such as PCSK9 and VEGF-B are under early investigation for their potential to provide combined metabolic and hemodynamic benefits.

### 10.3 Biomarker-driven patient selection

Biomarker-guided patient stratification will be essential for enriching trial populations and identifying individuals most likely to benefit from a given molecularly targeted therapy. Candidate biomarkers include urinary TGF-β activity, circulating PCSK9 levels, and suPAR (41, 42).

## 11 Recommended future clinical trial design

Future DKD trials of mAbs should incorporate several design features. First, population enrichment will be important, with priority given to patients with persistent albuminuria, rapid eGFR decline, or biomarker evidence of target activation despite optimized standard therapy. Second, endpoints should match the expected biological effect of the intervention; 2-year eGFR slope or composite kidney outcomes may be appropriate for disease-modifying therapies, whereas early UACR change may be useful mainly for interim futility assessment (43). Third, adaptive designs may help identify ineffective strategies earlier and reduce unnecessary exposure. Fourth, biomarker stratification using urinary TGF-β activity, suPAR levels, PCSK9 concentrations, or other target-specific markers may improve patient selection and interpretation of negative trials. Finally, combination strategies should be evaluated because mAbs are unlikely to replace RAAS blockade, SGLT2 inhibitors, finerenone, or GLP-1 receptor agonists, but may provide additive benefit in selected high-risk patients.

## 12 Conclusion

The development of therapeutic mAbs for DKD reflects the broader trajectory of precision medicine. Although early strategies targeting TGF-β and VEGF-B were unsuccessful, they provided important insights into the complexity of human DKD and the limitations of single-pathway intervention. More recently, PCSK9 mAbs have emerged as particularly promising candidates, supported by both a strong mechanistic rationale and encouraging real-world evidence suggesting not only lipid-lowering effects but also potential direct renoprotection. Meanwhile, novel targets such as αvβ8 integrin and SIRPα are advancing in preclinical research, offering the possibility of more localized pathway modulation with fewer systemic adverse effects. Going forward, integration of these emerging targets with advanced drug-delivery technologies and more rigorous clinical trial designs may help translate the precision of biologic therapies into meaningful clinical benefit for patients with DKD.
